# PRESIDENTIAL SYMPOSIUM: MULTIDISCIPLINARY EMERGING PERSPECTIVES ON BUILDING AND MAINTAINING NETWORKS IN AGING.

**DOI:** 10.1093/geroni/igz038.2065

**Published:** 2019-11-08

**Authors:** Darina V Petrovsky, Jamie N Justice

**Affiliations:** 1 University of Pennsylvania, Philadelphia, Pennsylvania, United States; 2 Wake Forest School of Medicine, Winston-Salem, North Carolina, United States

## Abstract

This ESPO Presidential Symposium features a multidisciplinary perspective and recent scientific advances made by early career researchers from each of the GSA scientific sections. They will provide examples of how their work is addressing ways to build and maintain networks in aging and gerontological workforce. These talks will span research on the age-associated transcriptional networks (Biological Sciences, Kulkarni), enhancing care for persons with dementia using a professional healthcare network (Health Sciences, Kovaleva), ways to maintain care networks in nursing home residents (Behavioral and Social Sciences, Kennedy), exploring the impact of social isolation in older adults on the Autism Spectrum (Social Research, Policy, and Practice, Waldron) and reflections on a project that linked aging education and student involvement within the aging network at the state level (Academy for Gerontology in Higher Education, Stephenson). These talks will demonstrate the diversity of aims, strategies, methodologies, and tools employed across disciplines. In addition, these early career researchers will share how they use networks in their own disciplines to advance their science with the goal of building an independent program of research. We will conclude with a discussion on ways to identify synergies across different fields and promote strategies for successful cross-discipline collaboration.

